# Thrombin activatable fibrinolysis inhibitor as a bleeding predictor in liver transplantation: a pilot observational study

**DOI:** 10.5935/0103-507X.20160031

**Published:** 2016

**Authors:** Wagner Luis Nedel, Edison Moraes Rodrigues Filho, Alessandro Comarú Pasqualotto

**Affiliations:** 1 Postgraduate Program in Hepatology, Universidade Federal de Ciências da Saúde - Porto Alegre (RS), Brazil.; 2 Irmandade Santa Casa de Misericórdia de Porto Alegre - Porto Alegre (RS), Brazil.

**Keywords:** Carboxipeptidase B2, Fibrinolysis, Liver transplantation, Hemorrhage, Fibrinogen, MELD

## Abstract

**Objective:**

To correlate the levels of thrombin activatable fibrinolysis inhibitor in the immediate postoperative period and at 24 hours postoperatively with the volume of intraoperative bleeding.

**Methods:**

Twenty-one patients allocated immediately before (elective or emergency) liver transplantation were analyzed. Blood samples were collected for thrombin activatable fibrinolysis inhibitor analysis at three different time points: immediately before liver transplantation (preoperative thrombin activatable fibrinolysis inhibitor), immediately after the surgical procedure (immediate postoperative thrombin activatable fibrinolysis inhibitor), and 24 hours after surgery (thrombin activatable fibrinolysis inhibitor 24 hours after surgery). The primary outcome of the study was to correlate the preoperative and immediate postoperative levels of thrombin activatable fibrinolysis inhibitor with intraoperative blood loss.

**Results:**

There was a correlation between the preoperative thrombin activatable fibrinolysis inhibitor levels and bleeding volume (ρ = -0.469; p = 0.05) but no correlation between the immediate postoperative thrombin activatable fibrinolysis inhibitor and bleeding volume (ρ = -0.062; p = 0.79). No variable included in the linear regression analysis (prehemoglobin, prefibrinogen and preoperative thrombin activatable fibrinolysis inhibitor) was a bleeding predictor. There was a similar trend in the variation between the levels of thrombin activatable fibrinolysis inhibitor at the three different time points and fibrinogen levels. Patients who died within 6 months (14.3%) showed decreased preoperative and immediate postoperative levels of thrombin activatable fibrinolysis compared with survivors (preoperative: 1.3 ± 0.15 versus 2.55 ± 0.53, p = 0.06; immediate postoperative: 1.2 ± 0.15 versus 2.5 ± 0.42, p = 0.007).

**Conclusion:**

There was a moderate correlation between preoperative thrombin activatable fibrinolysis inhibitor and intraoperative bleeding in liver transplantation patients, although the predictive role of this variable independent of other variables remains uncertain. Preoperative and immediate postoperative thrombin activatable fibrinolysis inhibitor levels may have a role in the survival prognosis of this population; however, this possibility requires confirmation in further studies with larger sample sizes.

## Abstract (pt)

**Objetivo:**

Correlacionar os níveis de *thrombin activatable fibrinolysis inhibitor* no pós-operatório imediato e com 24 horas de pós-operatório com o volume de sangramento tansoperatório.

**Métodos:**

Foram analisados vinte e um pacientes alocados imediatamente antes do transplante hepático (eletivo ou de urgência), com coleta de amostras sanguíneas para análise de *thrombin activatable fibrinolysis inhibitor* em três diferentes momentos: imediatamente antes do transplante hepático (*thrombin activatable fibrinolysis inhibitor* pré-operatório), imediatamente após o procedimento cirúrgico (*thrombin activatable fibrinolysis inhibitor* pós-operatório imediato) e após 24 horas do final da cirurgia (*thrombin activatable fibrinolysis inhibitor* 24 horas pós-operatório). O principal desfecho do estudo foi correlacionar os níveis de *thrombin activatable fibrinolysis inhibitor* pré-operatório e de *thrombin activatable fibrinolysis inhibitor* pós-operatório imediato com perda sanguínea no transoperatório.

**Resultados:**

Houve correlação entre *thrombin activatable fibrinolysis inhibitor* pré-operatório e o volume de sangramento (ρ = -0,469; p = 0,05), mas não de *thrombin activatable fibrinolysis inhibitor* pós-operatório imediato (ρ = -0,062; p = 0,79). Em análise de regressão linear, nenhuma das variáveis incluídas (hemoglobina pré, fibrinogênio pré e *thrombin activatable fibrinolysis inhibitor* pré-operatório) se mostrou preditor de sangramento. Houve tendência semelhante na variação entre os níveis de *thrombin activatable fibrinolysis inhibitor* durante os três diferentes momentos e os níveis de fibrinogênio. Pacientes que evoluíram a óbito em até 6 meses (14,3%) apresentaram níveis diminuídos de *thrombin activatable fibrinolysis inhibitor* pré-operatório e de *thrombin activatable fibrinolysis inhibitor* pós-operatório imediato, comparando-se aos sobreviventes (pré-operatório: 1,3 ± 0,15 *versus* 2,55 ± 0,53; p = 0,06; e pós-operatório imediato: 1,2 ± 0,15 *versus* 2,5 ± 0,42; p = 0,007).

**Conclusão:**

Houve correlação moderada entre *thrombin activatable fibrinolysis inhibitor* pré-operatório e o sangramento transoperatório em transplante hepático, porém seu papel preditivo independente de outras variáveis ainda permaneceu incerto. *Thrombin activatable fibrinolysis inhibitor* pré-operatório e pós-operatório imediato podem ter um papel na avaliação da sobrevida dessa população, necessitando-se confirmar em novos estudos, de maior tamanho amostral.

## INTRODUCTION

Blood product transfusion is a common event during orthotopic liver transplantation. It affects patient outcomes and the viability of the transplanted organ.^(1)^ In most studies, blood product transfusion is associated with increased mortality rates, increased risk of infections, and graft dysfunction.^(2)^ The main risk factors for bleeding and transfusion in liver transplantation are patient age, liver cirrhosis severity (evaluated using the model for end-stage liver disease [MELD]), preoperative hemoglobin level, anesthesia time, and preoperative fibrinogen levels.^(3)^ At the time of graft perfusion, there is an overall reduction in prothrombotic factors and coagulation that is associated with concomitant tissue plasminogen activator (tPA) production and fibrinolysis. This reduction can potentially increase the risk of bleeding,^(4)^ which is partly reversed by antifibrinolytic drugs.

Thrombin-activatable fibrinolysis inhibitor (TAFI), also known as procarboxipeptidase B or U, is a liver protein that acts as a fibrinolysis inhibitor. It is converted into its active form (TAFIa) by the thrombin-thrombomodulin complex, which decreases plasmin production and suppresses the fibrinolytic cascade.^(5)^ The serum levels of TAFI are substantially decreased in cirrhotic patients and can reach undetectable levels in patients with advanced hepatocellular disease^(6)^ due to impaired synthesis.^(7)^ Hyperfibrinolysis is a common finding among cirrhotic patients and may contribute to the increased bleeding tendency among these patients, which is a TAFI-dependent mechanism.^(8)^ Decreased TAFI levels are found in this population and have been identified as significant mortality predictors^(9)^ that are also related to cirrhosis severity.^(8)^ However, the role of TAFI in perioperative bleeding assessment and management in liver transplant recipients has not yet been established, which is the main objective of this study.

## METHODS

We studied 21 patients with liver cirrhosis of different etiologies who were undergoing perioperative management immediately before liver transplantation. Patients were admitted to the *Hospital Dom Vicente Scherer*, which has an 80-bed liver transplantation ward and is located in the south region of Brazil. Disease severity was evaluated using the MELD and Child-Pugh scores. Blood samples were collected for TAFI analysis using an enzyme-linked immunosorbent assay (ELISA; VisuLize TAFI antigen kit, Affinity Biologicals, Inc., Ancaster, Canada). Blood was collected into trisodium citrate vials, pH 7.5, from the antecubital vein at three different time points: immediately before liver transplantation (PRE TAFI), immediately after the surgical procedure (IPO TAFI), and 24 hours after surgery (TAFI 24 hours PO). These samples were analyzed according to the manufacturer's instructions, and the results are expressed in absolute values. Plasma was separated via centrifugation at 2,000 g for 15 minutes. Routine laboratory tests, including hematology tests, were performed according to the local perioperative protocols on fresh plasma samples, whereas the TAFI tests were performed on samples stored at -80°C for a maximum of 8 months. The absolute values of TAFI are expressed as µg/mL. Clinical characteristics and outcomes were analyzed via medical chart review.

This study was approved by the Research Ethics Committee of the institution (number 00796512.0.00005335), and the patients or their legal guardian signed an informed consent form. Exclusion criteria were age < 18 years, combined liver-kidney transplantation, liver retransplantation, and the use of vitamin K inhibitors, anticoagulants, or plasma transfusion up to 7 days before the surgical procedure. Primary outcome of the study was to correlate the PRE and IPO TAFI levels with intraoperative bleeding, defined as the volume measured by aspiration and using the cell saver system. Secondary outcomes were to correlate PRE TAFI levels with the different cirrhosis etiologies; to compare PRE TAFI levels with the presence of hepatocellular carcinoma or portal vein thrombosis; and to correlate TAFI levels with fibrinogen levels collected during the same period, the occurrence of shock 24 hours after transplantation, the need for surgical intervention within 48 hours of liver transplantation and the 6-month mortality rate.

### Statistical analysis

Values are expressed as the mean ± standard deviation (SD) or the median ± interquartile range depending on the distribution of values, which was assessed using the Shapiro-Wilk test for normal distribution. Continuous variables were analyzed using the Mann-Whitney test, and correlations between these variables were analyzed using Spearman's correlation test. Correlations between risk factors and perioperative bleeding were assessed using linear regression analysis, and variables were adjusted for age, gender, MELD, preoperative hemoglobin level, preoperative fibrinogen level, PRE TAFI, IPO TAFI and anesthesia time. Correlations between different TAFI and fibrinogen level time points were compared using generalized linear model, and the variation in mean values was compared using the Z score at each time point (preoperative, postoperative, and 24 hours postoperatively) for both variables. All statistical analyses were performed using the Statistical Package for the Social Sciences (SPSS), version 17.0, and STATA, version 11.0.

## RESULTS

A total of 26 patients were evaluated, but two patients failed to meet the inclusion criteria (combined liver-kidney transplantation), and three did not sign the informed consent form. The baseline patient data are summarized in table 1, and the main perioperative characteristics are shown in table 2.

**Table 1:** Clinical characteristics of the population

**Clinical characteristics**	
Gender (male)	17 (80.9)
Age	57 (9.2)
Donor age	49 (16.1)
Liver graft	
Pretransplant steatosis	4 (19)
Prior vasopressor use	14 (66.7)
Child-Pugh Score	
A	8 (38.1)
B	6 (28.6)
C	7 (33.3)
MELD	14 (7.1)
Etiology of cirrhosis	
Alcoholism	14 (66.6)
HCV	13 (61.9)
HBV	3 (14.9)
Primary sclerosing cholangitis/primary biliary cirrhosis	2 (9.6)
Cirrhosis complications	
Hepatopulmonary syndrome	1 (4.8)
Hepatorenal syndrome	4 (19.0)
Portal-systemic encephalopathy	2 (9.5)
Spontaneous bacterial peritonitis	9 (42.9)
Esophagogastric varices	4 (19.0)
Refractory ascites	11 (52.4)
Hepatocellular carcinoma	5 (23.8)
Portal vein thrombosis	5 (23.8)

MELD - Model for End-Stage Liver Disease; HCV - hepatitis C virus; HBV - hepatitis B virus. Results are expressed as number (%) and median (standard deviation).

**Table 2:** Perioperative data

**Variable**	
SOFA score upon ICU admission	9 (2.0)
Total perioperative bleeding (mL)	3940 (2608)
Anesthetic time (minutes)	355 (132)
Warm ischemia time (minutes)	67 (25)
Cold ischemia time (minutes)	407 (116)
Blood product transfusion	
Platelet concentrate	6 (28.6)
Packed red blood cells	12 (57.1)
Prothrombin complex concentrate	2 (9.5)
Cryoprecipitate	7 (33.3)
Antifibrinolytic administration	10 (47.6)

SOFA - Sequential Organ Failure Assessment; ICU - intensive care unit. Results are expressed as median (standard deviation) or number (%).

There was no significant difference between the PRE TAFI serum levels according to the different cirrhosis etiologies: alcoholism (p = 0.73), hepatitis C virus (p = 0.12), hepatitis B virus (p = 0.61), primary biliary cirrhosis (p = 0.18) and primary sclerosing cholangitis (p = 0.09). Patients with a diagnosis of portal vein thrombosis had higher TAFI serum levels than those who did not develop this complication: 2.8 ± 0.39 versus 2.0 ± 0.64 (p = 0.028), respectively. Patients with hepatocellular carcinoma also had higher PRE TAFI serum levels than those who did not have the disease: 2.7 ± 0.53 versus 2.0 ± 0.58 (p = 0.01).

TAFI levels varied over the three different time points in a manner that was not significantly different from the variation observed in fibrinogen during these three periods according to an analysis using the generalized linear model (Table 3). In the correlation analysis, the PRE TAFI levels showed correlations with the perioperative bleeding volume (ρ = -0.469; p = 0.05), the preoperative fibrinogen level (ρ = -0.603; p = 0.006) and the hemoglobin level (ρ = -0.630; p = 0.003) but not with the IPO TAFI levels (ρ = -0.062; p = 0.79). Linear regression showed that no variable included in the analysis was a significant predictor of perioperative bleeding after confounding variables were controlled (Table 4).

**Table 3:** Correlation between thrombin activatable fibrinolysis inhibitor and fibrinogen at different collection times

**Collection times**	**Mean difference between TAFI and fibrinogen**	**95%CI**	**p value**
Preoperative	0.31	-0.12 - 0.76	0.16
Immediate postoperative	0.12	-0.44 - 0.20	0.46
24 hours after surgery	0.08	-0.34 - 0.51	0.70

TAFI - thrombin activatable fibrinolysis inhibitor; 95%CI - 95% confidence interval.

**Table 4:** Risk factors predicting perioperative bleeding according to regression analysis

	**Mean ± SD**	**Crude linear regression**		**Adjusted linear regression**	
		**β (95%CI)**	**p value**	**β (95%CI)**	p value*
Pre TAFI	2.35 ± 0.67	-1.697.17 (-3.368.66 - -25.67)	0.047	-915.97 (-3.505.45 - 1.675.50)	0.463
Pre Fibr	187.10 ± 113.34	-12.35 (-22.50 - -2.21)	0.020	-4.99 (-17.73 - 7.75)	0.417
Pre Hb	12.25 ± 2.31	-684.08 (-1.112.98 - -255.18)	0.003	-348.78 (-1.009.79 - 312.21)	0.278

SD - standard deviation; 95%CI - 95% confidence interval; TAFI - thrombin activatable fibrinolysis inhibitor; Fibr - fibrinogen; Hb - hemoglobin. Sample size: 21 patients.

* Control performed between all variables.

There was no correlation between the occurrence of shock after the first 24 hours of the postoperative period and the PRE TAFI levels (2.1 ± 0.78 in patients with shock versus 2.4 ± 0.61 in patients who did not develop shock; p = 0.36) or the IPO TAFI levels (2.0 ± 0.69 in patients with shock versus 2.5 ± 0.44 in patients who did not develop shock; p = 0.16). The need for blood product transfusion in the first 24 hours of the postoperative period was also not correlated with the PRE TAFI levels (2.1 ± 0.70 in the transfusion group versus 2.45 ± 0.62 in the group without transfusion; p = 0.23) or the IPO TAFI levels (transfusion group 2.0 ± 0.69 versus 2.45 ± 0.50; p = 0.43) or with the need for surgical reintervention within 48 hours (PRE TAFI, reintervention group: 2.1 ± 0.65 versus 2.45 ± 0.67; p = 0.36; IPO TAFI, reintervention group: 2.0 ± 0.80 versus 2.45 ± 0.53; p = 0.39).

Within 6 months of follow-up, three patients died, for a total mortality rate of 14.3%. Patients who died had lower PRE TAFI (1.3 ± 0.15 versus 2.55 ± 0.53; p = 0.06, respectively) and IPO TAFI (1.2 ± 0.15 versus 2.5 ± 0.42; p = 0.007, respectively) serum levels than survivors, but there was no significant difference in the TAFI 24 hours PO levels between the groups (3.0 ± 0.92 among the survivors versus 2.8 ± 1.04 among the patients who died; p = 0.31).

There was no difference in the PRE TAFI and IPO TAFI levels between the patients who required transfusion in the first 24 hours post-surgery and those who did not (PRE TAFI: 2.1 ± 0.7 versus 2.45 ± 0.62; p = 0.43; IPO TAFI: 2.0 ± 0.69 versus 2.45 ± 0.50; p = 0.23), between the patients who developed persistent shock within 24 hours postsurgery and those who did not (PRE TAFI 2.5 ± 0.44 versus 2.0 ± 0.69; p = 0.16; IPO TAFI: 2.1 ± 0.78 versus 2.45 ± 0.61; p = 0.36), or between the patients who required surgical reintervention within 48 hours postsurgery and those who did not (PRE TAFI: 2.1 ± 0.65 versus 2.45 ± 0.67; p = 0.39; IPO TAFI: 2.0 ± 0.80 versus 2.45 ± 0.53; p = 0.36).

## DISCUSSION

This study reports key findings regarding TAFI among patients undergoing liver transplantation. The correlation between TAFI serum levels and the severity of chronic liver failure has been well described,^(8,9)^ although this is the first study to describe the variation in TAFI levels during the perioperative period and its correlation with different clinical outcomes. The relationship between the presence of portal vein thrombosis and increased TAFI levels must be highlighted. The literature suggests that a high TAFI level is a risk factor for venous thrombosis^(6,10,11)^ and is a finding in patients who develop disease recurrence.^(12)^ Patients with hepatocellular carcinoma may develop hypercoagulability, which causes decreased fibrinolytic activity during the intraoperative period of liver transplantation^(13)^ Our finding of increased TAFI levels among this population may corroborate such data.

Fibrinogen, a fibrin precursor, plays a key role in clot stabilization, providing resistance to fibrinolytic action. Serum levels are decreased during preoperative period because fibrinogen hepatic synthesis, and its serum level variation during the surgical procedure is mainly because of the degree of fibrinolysis present.^(1)^ The variation in TAFI levels at different time points follows the variation in fibrinogen levels, which corroborates such findings.

Prothrombin time, which is a component of the Child-Pugh and MELD (international normalized ratio - INR) scores, is an exception. The determination of individual coagulation factors adds little to the prognosis of patients with chronic liver failure. A previous study of cirrhotic patients found that TAFI serum level is mortality predictor.^(9)^ However, the present study found a correlation between mortality and preoperative and immediate postoperative liver transplantation TAFI levels. This finding may be explained by the metabolic function of both the cirrhotic liver and the transplanted liver because increased fibrinolytic activity is not found among non-survivors.^(9)^

Currently, most studies fail to define risk factors related to bleeding, including laboratory tests.^(14,15)^ Few data reported in the literature suggest that MELD score, preoperative hemoglobin levels, preoperative fibrinogen serum levels, and anesthesia time are predictors of increased bleeding during transplantation.^(3)^ Hemostatic abnormalities during the perioperative period of liver transplantation are multifactorial, and only a few laboratory tests are accurate predictors of this outcome. In this study, we found only a moderate correlation between PRE TAFI and bleeding volume, and this correlation was lower than the correlation between fibrinogen and preoperative hemoglobin levels. In an adjusted linear regression model, none of these changes were found to predict bleeding during liver transplantation, although the small sample size precludes definitive conclusions.

This study has several limitations. First, this is merely a hypothesis-generating pilot study that addresses issues that should be further examined in a larger sample and with better methodological quality. Our results preclude making conclusive statements regarding mortality prediction in this population because data could not undergo multivariate analysis given the low incidence of deaths.

The failure to compare the role of TAFI as a bleeding predictor with thromboelastography, the preferred test for this purpose, is a key caveat of this study. Thromboelastography enables the accurate and immediate determination of the etiology of bleeding (fibrinolysis, mechanical causes or thrombocytopenia) in a dynamic assessment, thereby decreasing the need for blood product transfusion in liver transplantation.^(16)^ Comparing TAFI levels and their variations (delta) among different time points with thromboelastography findings or other fibrinolysis markers (e.g., fibrinolysis time and clot lysis time) would more accurately establish the correlation between TAFI and the presence of fibrinolytic activation during the perioperative period.

## CONCLUSION

Thrombin activatable fibrinolysis inhibitor, particularly its preoperative levels but also its levels during the immediate postoperative period, may be a key tool for further understanding coagulopathy during liver transplantation, and it may have a role as a predictor of increased mortality rates in the liver transplant population. Further studies with a larger sample size that measure thrombin activatable fibrinolysis inhibitor levels beyond the first 24 hours after surgery are needed to establish the role of thrombin activatable fibrinolysis inhibitor in pre-liver transplantation stratification.
